# Identification of animal movement patterns using tri-axial magnetometry

**DOI:** 10.1186/s40462-017-0097-x

**Published:** 2017-03-27

**Authors:** Hannah J. Williams, Mark D. Holton, Emily L. C. Shepard, Nicola Largey, Brad Norman, Peter G. Ryan, Olivier Duriez, Michael Scantlebury, Flavio Quintana, Elizabeth A. Magowan, Nikki J. Marks, Abdulaziz N. Alagaili, Nigel C. Bennett, Rory P. Wilson

**Affiliations:** 1 Department of Biosciences, College of Science, Swansea University, Swansea, SA2 8PP UK; 2 ECOCEAN Inc. (Aust.), ECOCEAN (USA), C/o 68a Railway Street, Perth, WA 6011 Australia; 3 FitzPatrick Institute of African Ornithology, DST-NRF Centre of Excellence, University of Cape Town, Rondebosch, 7701 South Africa; 4 CEFE UMR 5175, CNRS, Université de Montpellier, Université Paul-Valéry Montpellier, EPHE, 1919 route de Mende, 34293 Montpellier Cedex 5, France; 5 School of Biological Sciences, Institute for Global Food Security, Queen’s University Belfast, Belfast, Ireland; 6 Centro Nacional Patagónico, CONICET (9120), Puerto Madryn, Chubut Argentina; 7 KSU Mammals Research Chair, Department of Zoology, College of Science, King Saud University, P.O. Box 2455, Riyadh, 11451 Saudi Arabia; 8 Department of Zoology and Entomology, University of Pretoria, Pretoria, South Africa

**Keywords:** Magnetometer, Magnetic field, Bio-logging, Accelerometer, Animal behaviour, Behavioural consistency, Normal operational plane, Angular velocity

## Abstract

**Background:**

Accelerometers are powerful sensors in many bio-logging devices, and are increasingly allowing researchers to investigate the performance, behaviour, energy expenditure and even state, of free-living animals. Another sensor commonly used in animal-attached loggers is the magnetometer, which has been primarily used in dead-reckoning or inertial measurement tags, but little outside that. We examine the potential of magnetometers for helping elucidate the behaviour of animals in a manner analogous to, but very different from, accelerometers. The particular responses of magnetometers to movement means that there are instances when they can resolve behaviours that are not easily perceived using accelerometers.

**Methods:**

We calibrated the tri-axial magnetometer to rotations in each axis of movement and constructed 3-dimensional plots to inspect these stylised movements. Using the tri-axial data of Daily Diary tags, attached to individuals of number of animal species as they perform different behaviours, we used these 3-d plots to develop a framework with which tri-axial magnetometry data can be examined and introduce metrics that should help quantify movement and behaviour.﻿﻿

**Results:**

Tri-axial magnetometry data reveal patterns in movement at various scales of rotation that are not always evident in acceleration data. Some of these patterns may be obscure until visualised in 3D space as tri-axial spherical plots (*m-spheres*). A tag-fitted animal that rotates in heading while adopting a constant body attitude produces a ring of data around the pole of the *m-sphere* that we define as its Normal Operational Plane (NOP). Data that do not lie on this ring are created by postural rotations of the animal as it pitches and/or rolls. Consequently, stereotyped behaviours appear as specific trajectories on the sphere (*m-prints*), reflecting conserved sequences of postural changes (and/or angular velocities), which result from the precise relationship between body attitude and heading. This novel approach shows promise for helping researchers to identify and quantify behaviours in terms of animal body posture, including heading.

**Conclusion:**

Magnetometer-based techniques and metrics can enhance our capacity to identify and examine animal behaviour, either as a technique used alone, or one that is complementary to tri-axial accelerometry.

**Electronic supplementary material:**

The online version of this article (doi:10.1186/s40462-017-0097-x) contains supplementary material, which is available to authorized users.

## Background

Animals behave in ways to enhance their lifetime fitness, choosing from their behavioural repertoire according to their environmental and internal circumstance [1]. Thus, behaviour is at the root of basic and applied ecology and its study is pivotal to understanding individual, community and ecosystem processes. Techniques used to study and quantify animal behaviour have gone beyond direct observation to an increasing use of diverse animal-attached logging devices (e.g. GPS or light-level loggers [2, 3]). In particular, the last decade has seen widespread use of accelerometers to quantify metrics relating to behaviour (e.g. [4, 5]). This is because most behaviours are defined by movements and/or postural patterns, both of which can be quantified using accelerometers (see [6, 7]). Specifically, orthogonally-orientated tri-axial accelerometers can provide high resolution (infra-second) data to define tag-orientation with respect to gravity (if no other forces are operating), and therefore animal posture (the ‘static’ acceleration component) [6, 8], as well as the extent of movement given by the dynamic component of acceleration [4, 6]. As a result, accelerometers in animal-attached tags are now in widespread use and are acknowledged as being an extremely powerful methodology for elucidating animal behaviour [7]. Beyond this, the dynamic acceleration has also been shown to be a powerful proxy for movement-based energy expenditure [9–12], providing yet further information valuable for understanding behavioural processes.

However, not all behaviours are well described by accelerometers. Perhaps most importantly, accelerometers alone cannot resolve animal heading. Interpretation of accelerometer-informed movement can also be confounded by forces not generated by the animal themselves, such as the motion of waves for birds resting on the sea surface (*cf*. [13]) or vibration due to air-flow, increasing the signal-to-noise ratio in the data (*cf.* [14]). In addition, a particular problem arises when animal velocity is constant and there is little or no animal-induced acceleration, such as occurs in marine and aerial species during gliding (e.g. [15]) or when body parts move slowly, at constant velocity. Finally, although animal postural data, which is an important component in helping define behaviour [6], can generally be derived by applying a high-pass filter or by smoothing the acceleration data, this breaks down when, animals ‘pull g’, such as when a cheetah corners fast (e.g. [16]) or a bird banks or dives sharply [17].

Many of these problems can be resolved, however, by using other tri-axial sensors measuring angular rotation, notably gyroscopes or magnetometers [18], both of which are often combined with accelerometers in inertial measurement units (e.g. [19, 20]). Gyroscopes measure angular velocity, and are very sensitive to angular rotation although they are subject to drift over time [21]. Magnetometers do not drift and can be used to derive heading (generally in association with accelerometers, but see below) but produce signals that are complex to interpret because the output varies with location on the earth (*cf*. [22]) and there are some particular instances when magnetometers are insensitive to angular rotation (see later).

This work considers the potential of magnetometers in animal-attached tags to determine behaviour in a manner analogous to accelerometers. Magnetometers are not sensitive to acceleration of any sort (gravitational or dynamic) and so can be used in tandem with accelerometers to enhance the acquisition of metrics useful for quantifying animal behaviour [18, 19, 23]. Indeed, the simultaneous use of these two sensor types enables animal heading to be resolved, which provides extraordinarily finely resolved animal trajectories if the data are dead-reckoned with appropriate temporal resolution [22, 24]. Magnetometers react to magnetic field orientation and intensity in a manner analogous to the response of accelerometers to gravity, and thus there is also the potential for them to act as movement sensors, although their reaction characteristics differ fundamentally to those of an accelerometer.

Magnetometers have developed from relatively insensitive, single-axis sensors that measure proximity to a magnet, based on local magnetic field strength, to tri-axial sensors that are capable of recording orientation in relation to the Earth’s magnetic field (hereafter referred to as TriMag sensors). In early work, mono-axial magnetometers were used to document the general activity of a loggerhead turtle (*Caretta caretta*) by sensing the position of a strong magnet within a compass [25], and further applications of mono-axial sensors have primarily concentrated on measuring the proximity between an animal-attached tag and a magnet, mounted on some moving part of the animal [26, 27]. Thus, for example, this approach has been used to quantify limb movement in swimming animals [26] as well as mandible movement to quantify the feeding and breathing behaviour of free-living animals [27, 28]. However, the extreme sensitivity of modern magnetometers now allows magnetic field-sensing transducers to define the Earth’s magnetic field intensity in all three spatial dimensions and therefore resolve angular rotations with 1–2° of accuracy (*cf.* [29–31]).

Given this sensitivity, there is great potential for such systems to help elucidate animal behaviour based on angular rotation. With the current trend being towards longer deployments of animal-attached tags, the TriMag sensor *per* se has increasing value in quantifying angular rotation. Although, to date, magnetometers have been little used in studies of behaviour in this regard (but see [18]) excepting the notable, and increasingly rich, literature of them being used in tandem with gyros and/or accelerometers to dead-reckon (e.g. [29, 32–34]).

Here, we test the use of the tri-axial magnetometer as a sensor to resolve animal movement, paying particular attention to cases where it can provide further information beyond that which can be achieved with accelerometers. We first compare accelerometer and magnetometer data recorded from devices attached to different free-living animals, to highlight the types of movement that can be recorded by the two types of transducer. Second, we provide a method of calibrating the TriMag sensor to the Earth’s magnetic field according to the normal orientation of the carrier animal. Third, we go on to propose the use of tri-axial plots to help interpret TriMag data, following the utility of this approach for other orthogonally mounted sensor data [35]. This approach visualises the data and allows angular rotations to be easily resolved. Finally, we derive several metrics from these 3-dimensional data and examine how they can be best interpreted to maximise the information obtainable, focusing on movement consistency and performance. Our general aim is to make researchers who are already familiar with tri-axial acceleration data aware of the strengths and weaknesses of TriMag data for studying animal behaviour, and to provide a framework within which they can work to identify patterns that might otherwise be hidden.

## Methods

### Background to the general output of magnetometry sensors

The Earth’s magnetic field is often envisaged as field lines that run from the magnetic north to the magnetic south, with the lines being perpendicular to the north–south axis at the magnetic poles, horizontal to it at the magnetic Equator and at an angle of declination to the Earth’s surface for areas in between. Magnetometers measure magnetic field intensity, with a single-axis sensor producing a maximum value when it faces magnetic north and has its measurement axis exactly parallel to the magnetic field lines, and a minimum value when rotated through 180° so that it is facing magnetic south. The output values generated as the magnetometer rotates from north to south follow a sine wave, with an exactly intermediate value between the maxima when the magnetometer is orientated perpendicular to the Earth’s magnetic field.

The combined outputs of three orthogonally mounted magnetometers together define the orientation of the TriMag sensor with respect to the Earth’s magnetic field. The data thus allow the orientation of a tag-bearing animal to be defined with respect to the Earth’s magnetic field. The only exception is where one of the TriMag axes is aligned with the inclination lines. In this case, rotation of the magnetometer about the aligned axis produces no change in any of the axes outputs and is essentially a ‘blind spot’ to instantaneous angular rotation. Such a scenario is, however, unlikely during normal animal movement, especially over long periods of time, as animals generally move in-line with the Earth’s surface, which differs to the plane of the magnetic field lines (see above). Indeed, this applies to most locomotory behaviour in most animals on Earth, with the exception of those at the magnetic poles, where inclination lines are perpendicular to the Earth’s surface. For example, in the only study of its kind, Martín López et al. [18] found for a Blainville’s beaked whale (*Mesoplodon densirostris*) tagged in Spain, only ~0.2% of typical diving behaviour was aligned in the manner described above, with the inclination lines of the magnetic field.

### Data collection

We examined TriMag data recorded using Daily Diary (DD) logging units [23], deployed for a variety of reasons but not specifically for consideration of the TriMag data as documented here. The DD devices weighed between approximately 16 and 60 g, with dimensions from 33 × 38 × 10 mm to 80 × 37 × 25 mm, depending on the battery and housing design. These devices were attached to free-living vertebrates (a fish, 3 bird and 3 mammal species) and aspects of their behaviour analysed through comparison of accelerometry and magnetometry data. In all cases presented here, we refer to the x, y and z axes of the magnetometer, which correspond to the dorso-ventral, lateral and longitudinal axes of the animal, respectively. Orthogonal TriMag data were recorded in gauss (G) using the ultra-compact high-performance eCompass module consisting of a combined 3D digital linear accelerometer and ST digital magnetometer (LSM303DLHC, STMicroelectronics www.st.com). This sensor recorded the magnetic intensity within the range of −1.3 to + 1.3 G at 16-bit data-output resolution. The DDs recorded tri-axial magnetometer data, as well as tri-axial acceleration (*g*), barometric pressure (Pa) and temperature (°C), all at 8–40 Hz (see [23] and relevant sections of text below). Data were stored on an on-board 2 Gb memory card.

To aid comprehension of TriMag data, we used defined movement protocols to calibrate the system with respect to the angular rotation that occurs when an animal changes (i) posture and (ii) heading. Following these tests, we quantified the movement of free-living animals according to the frequency distributions of rotational data as well as the centroid and angular velocity within the specific trajectory of rotation that is associated with different behaviours. The calibration protocols and study species are described in detail on presentation of the data.

## Results and discussion

### Comparison of accelerometer and TriMag sensor data

**Fig. 1:**
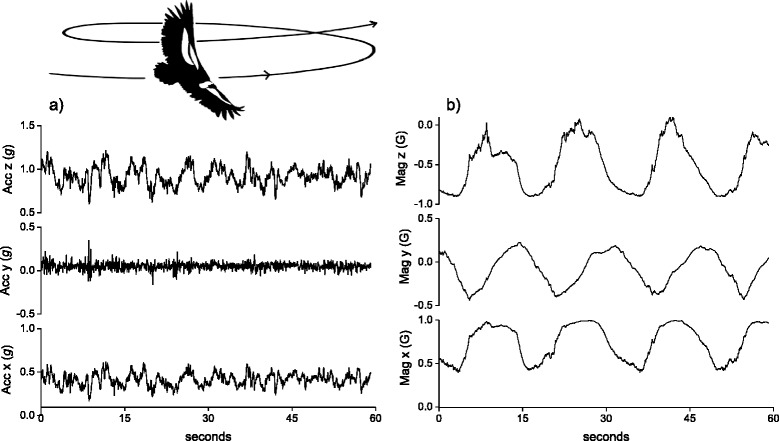
A time series plot of 40 Hz tri-axial acceleration data (**a**) and tri-axial magnetometry data (**b**) of a Himalayan griffon vulture (*Gyps himalayensis*) soaring in a thermal updraught in France (a hand-tame captive individual). The bird was wearing a tag that was attached to the its back using Velcro onto a baseplate fixed using a leg loop harness (see [53] for details)

The first movement protocol relates to changes in the animal’s yaw axis only and for this we use thermal soaring, where birds move at near constant speed with little or no limb movement and adopt a certain bank angle within a rising column of warm air [36]. Figure 1 shows a 60 s plot of tri-axial acceleration data (Fig. 1a) alongside TriMag data (Fig. 1b) for a Himalayan vulture (*Gyps himalayensis*) soaring in a thermal updraught (collected at 40 Hz). The magnetometry data, unlike the acceleration data, clearly display the circling motion of the soaring vulture in the sine wave pattern of each TriMag axis [15]). Thermal soaring is evident in each axis due to the change in heading as the bird circles, and thus TriMag data provide a signature of a particular movement that relates to exploitation of environmental energy. Because there is little change in speed of the soaring vulture, the changes in acceleration data mainly reflect turbulence and small postural changes undergone by the bird during circling but provide no definitive signal for thermal soaring in itself [15].

**Fig. 2:**
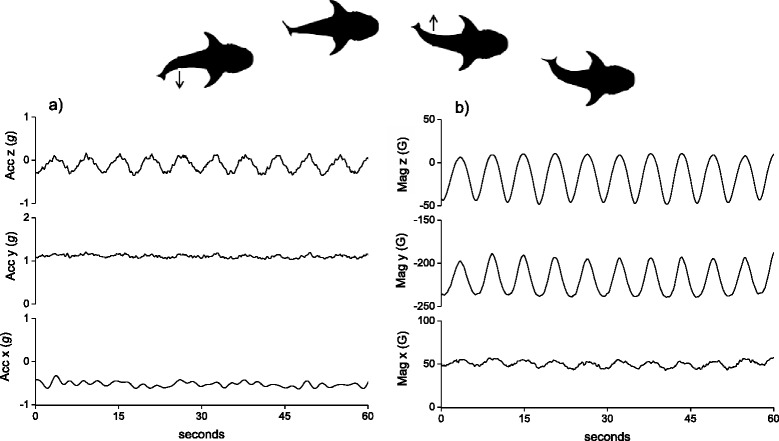
A time series plot of 6 Hz tri-axial acceleration (**a**) data and its associated tri-axial magnetometry data (**b**) in a swimming whale shark (*Rhincodon typus*) (equipped in Australia). The tag was attached to the second dorsal fin of the fish using a clamp system (see [54])

The second movement protocol illustrates how sensor data may incur noise due to the manner in which the tag is mounted on an animal. Our case is swimming by a whale shark (*Rhincodon typus*) with a DD mounted near the tail (recording at 6 Hz). Figure 2 shows a 60 s plot of tri-axial acceleration data (Fig. 2a) with TriMag data (Fig. 2b) with a reasonably clear oscillating pattern in the acceleration data, predominantly in the sway axis (Fig. 2a), due to the changing velocity with each tail stroke. The stroke frequency is, however, relatively low (<1 Hz; *cf*. [37]) so the acceleration signal is weak. This is further blurred by noise in dynamic acceleration that is mostly due to translocational movement of the tag, likely stemming from the movement of water past the fin and tag, causing them to flutter. Such ‘noise’ in the acceleration data is also apparent when tags are attached to birds’ feathers with tape (*cf.* Fig. 1a) [38] where the tape does not provide a fully stable attachment. Magnetometers, on the other hand, are unaffected by gravity and dynamic, translocational movement since they only record angular rotation. The magnetometer output is therefore generally less sensitive to this noise (Fig. 2). It is this low signal-to-noise ratio and insensitivity to dynamic movements that particularly lends the sensor to investigations of body, or body part, rotation. For example, Martín López et al. [18] use the smooth signal of a magnetometer to characterise swimming gaits in diving marine mammals according to stroke durations, and go on to subtract its values of body rotation to partition the acceleration signal into gravitational acceleration (body rotation) and dynamic acceleration (or specific acceleration) [18].

**Fig. 3:**
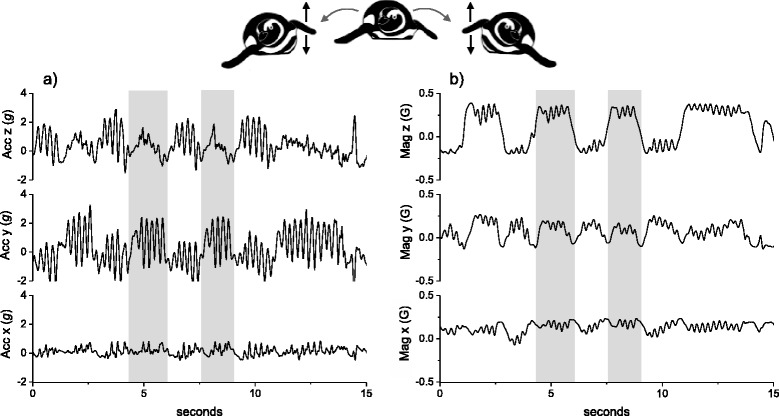
A time series plot of 40 Hz tri-axial acceleration data (**a**) with its associated tri-axial magnetometry data (**b**) of a washing Magellanic penguin (*Spheniscus magellanicus*) (equipped in Argentina *cf.* [55]). The tag was attached to the bird’s back with tape [38]

The third movement protocol illustrates that rotational movement in almost any axis, not just the yaw plane (see above), is well defined by TriMag data. The example is washing at sea by a Magellanic penguin (*Spheniscus magellanicus*), which consists of a highly conserved movement pattern in which birds roll to one side and then the other, while rubbing their flanks with their flippers. Figure 3 demonstrates that some postural movements, particularly the roll of the bird (Fig. 3a), are visible in the tri-axial accelerometer data but are also clearly identifiable in the TriMag data (Fig. 3b) (with 40 Hz data collection). Indeed, although the acceleration data show obvious increased activity, the changes in body roll are less readily resolved (Fig. 3a) than in the TriMag data because the dynamic acceleration masks the static acceleration change. For acceleration-only tags, this would normally be dealt with by deriving the static acceleration. On first inspection though, the TriMag data immediately show the postural angular rotation associated with both the major body rolls (at a frequency of *ca* 0.3 Hz) and the flipper movement (at a frequency of *ca* 2 Hz) (Fig. 3b), without interference from dynamic movement.

We used a template-matching procedure (using Framework4 software as detailed in [39]) to quantify the potential value of TriMag and acceleration signals to detect and correctly classify the three behaviours described above. Using the TriMag template, more instances of the specific behaviours were detected than could be found with the acceleration equivalent template. Thus, for the Himalayan vulture, the TriMag signal correctly identified 32 of 63 cases of thermal soaring (51%) while the acceleration signal found 0 (0%). In the swimming instance, TriMag identified 417 of 445 cases (94%) while acceleration found 1 (0%). Finally, for the penguin, the TriMag found washing for 32 of 42 instances (76%) while the acceleration found 18 (43%) (for details and full precision documentation see Additional file 1). Overall, therefore, these examples show that angular rotation, as recorded by the TriMag sensor, can be a clear indicator of behaviour, manifest by directional changes over a range of timescales.

### Calibration of TriMag sensors and movement protocols

**Fig. 4:**
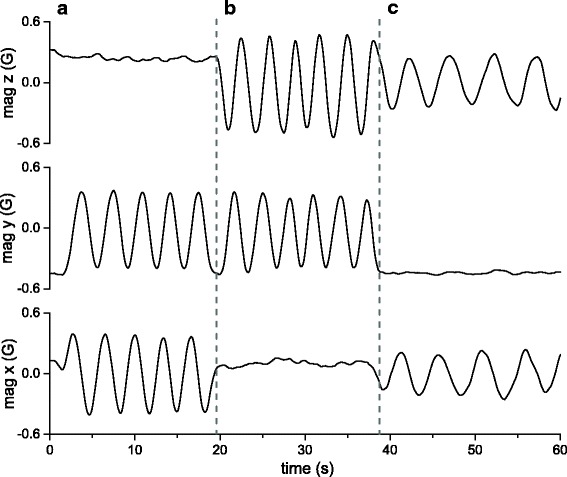
Calibration rotations through 360° about each of the TriMag axes and shown as the usual time-based plots. Rotations of the tag on its roll (**a**), pitch (**b**) and yaw (**c**) axes affect the outputs of two magnetometer axes to differing extents

As with tri-axial accelerometer and gyroscope measurements, the sensor outputs for each of the three tri-axial magnetometer axes may relate to the outputs of the other two. This means that, in order to obtain the complete picture of TriMag rotational movement, users should consider all three TriMag channels simultaneously. A standard calibration of a device containing TriMag sensors might be to hold the tag horizontal, facing North, and then rotate the unit through 360° in the yaw, then the pitch, then the roll axis. This would then effectively allow production of three graphs, each of time *versus* magnetic field strength (one for each sensor), showing varying response sine waves of the three axes depending on the magnetic declination (Fig. 4). The inter-relation of the three axes, however, makes it difficult to translate these calibration movements to help interpret data derived from a free-living animal where pitch, roll and yaw may be changing substantially.

### Tri-axial plots for interpretation of TriMag sensor data

#### Construction of the m-sphere

**Fig. 5:**
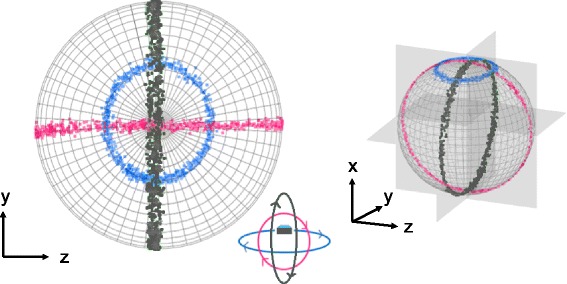
Calibration rotations through 360° about each of the TriMag axes (*cf.* Fig. 4), as visualised on the *m-sphere*. In this trial, multiple rotations were undertaken about each axis and are shown by lines of points in the circles created, coloured by each axis (*blue* = yaw, *red* = roll, *dark grey* = pitch). Variation in the position of points within a single rotational ring, are due to wobble in the rotating arm on which the tag was mounted

Here, we suggest and develop a framework that allows researchers to distinguish rotation in heading from that of posture based on the visualisation and interpretation of 3-dimentional plots. The nature of the TriMag outputs means that, when plotted in a tri-axial space, data lie on the surface of a sphere, which we term the ‘*m-sphere*’ (Fig. 5). This is because the vectorial sum of the magnetometry data is a constant, which is projected onto a tri-axial frame of reference. Indeed, given the tri-axial and orthogonal properties of accelerometer and magnetometer data, it makes sense for both data types to be visualised in tri-axial space. Wilson et al. [35] demonstrate the use of the *g-sphere* to help interpret accelerometry data illustrating variation in posture and we adapt this approach. It is important to note, however, that the interpretation of *g-spheres* and *m-spheres* differs fundamentally, due to their different frames of reference and the insensitivity of TriMag sensors to dynamic acceleration. The vector sum of the TriMag outputs is constant for any particular place on Earth, which should make the *m-sphere* perfectly spherical (although different places on the Earth’s surface may have *m-sphere*s of different diameter due to local or regional variations in magnetic field intensity). Ferrous material associated with the tag however, may distort the local magnetic field. This can cause the sphere to become displaced from its nominal centre (due to a hard iron distortion) or adopt a more ovoid form (soft iron distortion); both these distortions can be readily corrected using processes described by Bidder et al. [22] to centre the data around a sphere and normalise it to the surface of the sphere, respectively. We have made these corrections for data included here.

Plotting the simple stylised movements of the TriMag sensor calibration (see above) on the surface of the *m-sphere* maps clearly defined ring-shaped trajectories on the sphere (Fig. 5). Rotations in the conventional ‘yaw plane’, parallel to the surface of the Earth, map a circle on the *m-sphere* (Fig. 5, blue data points), whereas rotations through the pitch and roll planes map *great circle* rings from pole to pole at an orthogonal crossing point (Fig. 5, red and dark grey rings).

**Fig. 6:**
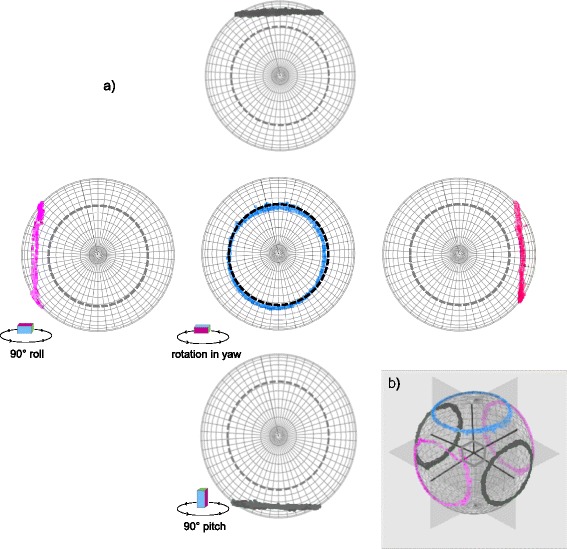
**a** The complete rotation of a DD with TriMag sensor about its yaw axes on a level surface (*blue circle; centre*) creates the Normal Operational Plane (NOP, *dashed outline*). The tag was tilted −90° and 90° in both its pitch and roll axes and the full yaw rotation repeated (*cf.* Fig. 4). This shows how the specific pattern of data on the *m-sphere*, or the ‘*m-print*’ of the behaviour, here created by a rotation, moves across the surface of the *m-sphere* as posture is altered (dark grey *m-print* rings correspond to rotations in pitch postures; pink *m-print* rings correspond to rotations in roll postures). **b** The centres of these rings on the surface of the m-sphere are 90° from the NOP corresponding to the 90° tilts in posture

In fact, any rotation in the yaw produces a ring on the *m-sphere,* but the position of the ring is determined by posture (Fig. 6). If the calibration procedure adopted above is extended to perform the rotation in yaw with the tag at 90° increments in pitch or roll from the start position relative to horizontal, the *m-sphere* will show a ring associated with the normal yaw rotation and four additional rings for each of the 90° postural rotations (Fig. 6).

If a tag containing a TriMag is mounted on an animal that normally operates in a particular posture, such as a walking or standing deer, full directional rotation of that animal in heading (i.e. yaw) will create a ring on the surface of the *m-sphere* (in the same manner as described above, Fig. 5, blue ring, Fig. 6, centre). We define this ring as the Normal Operational Plane (NOP) since it relates to the normal operational posture adopted by the animal when operating on the plane parallel to the Earth’s surface (i.e. yaw plane). Any deviation from the NOP on the surface of the *m-sphere* is therefore the result of change in animal pitch and/or roll. The position of the NOP itself will vary according to both the inclination angle of the magnetic field at that location, and the manner in which the tag has been attached to the animal. However, data on the *m-sphere* can be normalised *post-hoc* to reposition the NOP about the pole of the sphere on a horizontal plane to aid visual interpretation (see below) and essentially calibrate the tag to the animal’s movement. The only circumstance where directional rotation of the animal will not clearly define a NOP is at Earth’s magnetic poles because magnetic field runs perpendicular to the Earth’s surface.

#### M-sphere outputs for interpreting the behaviour of free-living animals

**Fig. 7:**
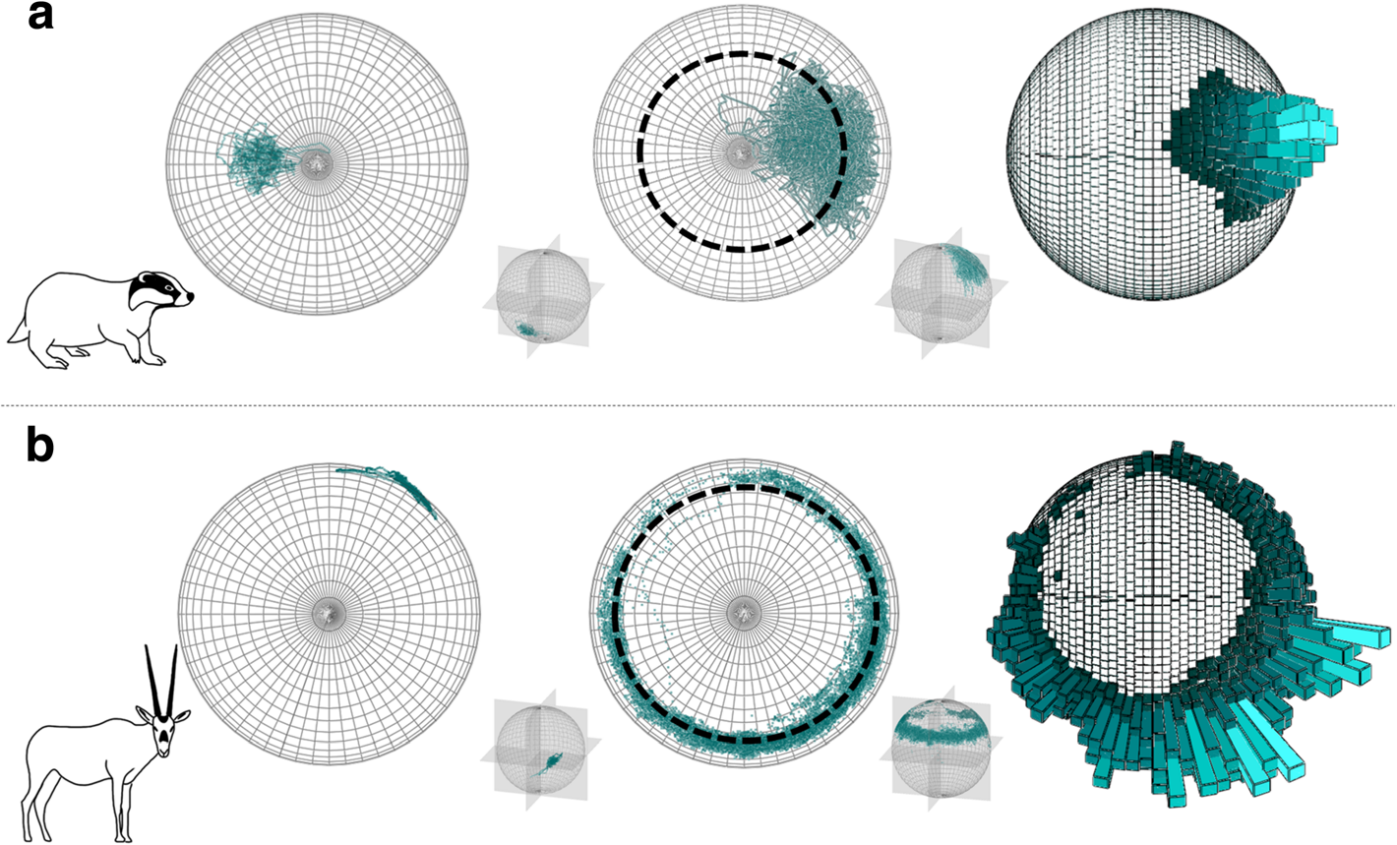
Tri-axial plots of 10 min of 40 Hz acceleration data (*left hand spheres*) and TriMag data (*middle and right hand spheres*) from (**a**) an active European badger (*Meles meles*) (equipped in Northern Ireland with a collar [56]), and (**b**) an Arabian oryx (*Oryx leucoryx*) (equipped in Saudi Arabia, again using a collar, Rees-Roderick *et al*. in preparation). The *g-spheres* (*left*) are 3-dimensional plots of static acceleration data, where the distribution of points illustrates the range in postural orientation of the animal in 3D space (see [35]). By comparison, the *m-spheres* (*middle*) also show postural information in angular rotation, but reveal how posture varies according to heading. Distribution of data about the NOP (*black dashed circle*) may be due to variation in postural rotation about a single heading, as is the case for the badger, or by directional rotation in the yaw axes, as seen in the oryx. The frequency distribution of TriMag data (*right*) highlights the predominant posture or direction associated with the behaviour

Plotting TriMag data on *m-spheres* highlights patterns in heading and postural rotation that are not obvious either by the consideration of acceleration data, or absolute values of heading. For example, examination of 10 min of randomly selected periods of active movement data for a European badger (*Meles meles*) and an Arabian oryx (*Oryx leucoryx*) show highly variable, species-specific *m-sphere* coverage. Some postural variation is evident in the acceleration data (Fig. 7, left), but the *m-spheres* (Fig. 7, middle) reveal very different patterns of movement for the two species. The badger data (collected at 20 Hz) lie about a specific region of the NOP (Fig. 7a, dashed ring, drawn from calibration of the tag prior to deployment), indicating firstly, that the heading of the animal was fairly invariant for this period, and secondly, since the radial spread of data about this section of the NOP is relatively large, that the body posture in terms of roll and pitch varied appreciably for any given heading (*cf.* Fig. 5). This is apparent even without consultation with the acceleration data because tri-axial plotted data that remain on the NOP correspond to no departure from the horizontal orientation. In comparison, the oryx displayed a comparatively well-defined, thin ring about the NOP*,* indicating relatively little change in body posture compared to the extensive variation in heading (but see below for discussion of the small variation in this).

This approach can be expanded to illustrate how animals apportion their time to different orientations, by constructing spherical histograms (Fig. 7, right) representing the density of points on the *m-sphere* (*cf.* (35)). In the cases examined above, the frequency distribution for the badger shows that the animal maintained a constant heading (Fig. 7a. right) with some variation in pitch and roll (Fig. 7a. middle). The oryx had a much greater range in heading than seen in the badger, almost filling the entire ring of the NOP (Fig. 7b. middle), albeit with some bimodality in heading (Fig. 7b. right). Both modes also show a greater spread of postural variation than other headings about the NOP, most likely indicating both ‘head-down’, i.e. grazing, and ‘head-up’ behaviour at these sites (the tags were collar-mounted and thus sensitive to neck angle).

The combination of heading and postural rotation provided by TriMag data, allows behaviour to be distinguished by simultaneous consideration of the two. This enhances biological interpretation of logger data beyond that possible with tri-axial acceleration data alone. For example, animal heading may be associated with particular postures, if for instance birds coming in to land recapitulate their landing trajectory, or where a ‘head-up’ or vigilant posture occurs at certain locations with patterns in directionality. In multiple associated tagged animals, this approach could quantify ‘information gathering’ via scanning behaviour or the cohesive movement of social groups, for example, where individuals make decisions on who to follow. The general omission of heading in behavioural texts to date may be largely due to the difficulties of resolving it, especially when tri-axial data is interpreted using 3 separate time-series plots.

**Fig. 8:**
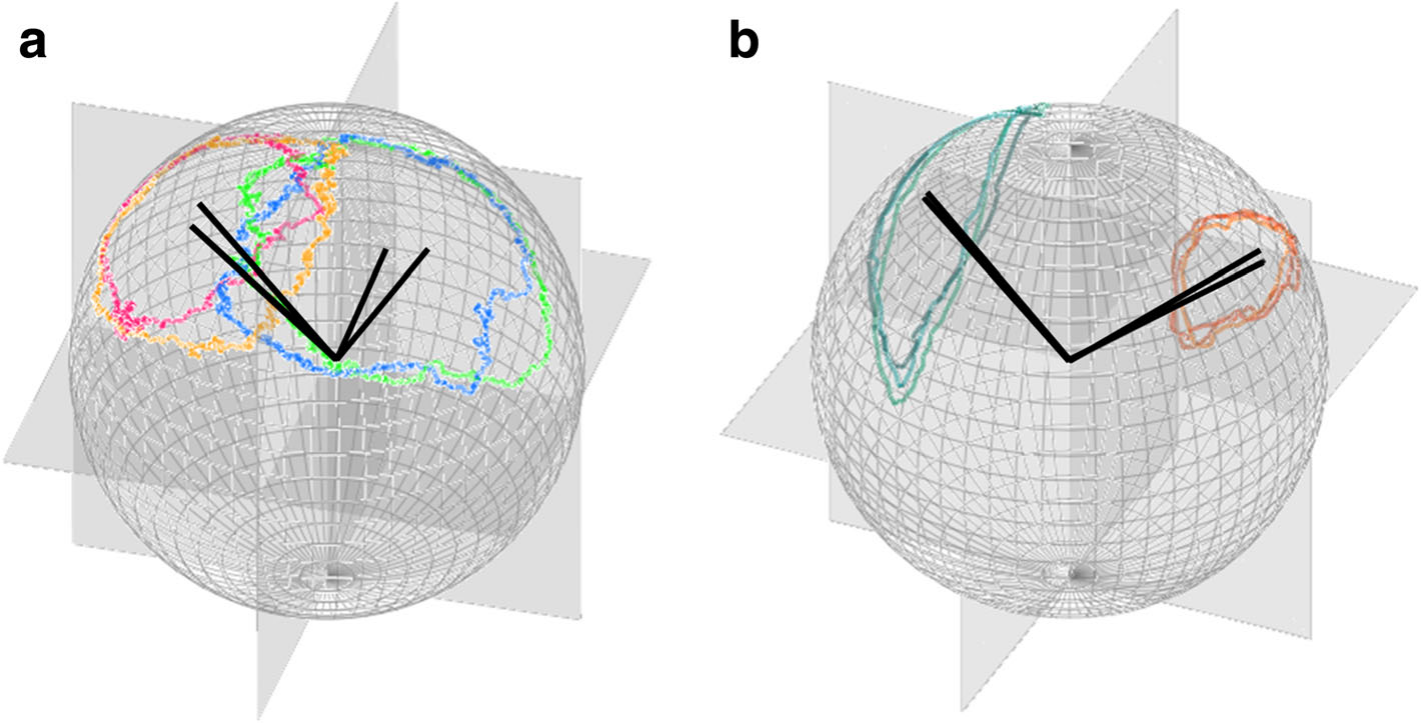
The specific *m-prints* of (**a**) the ring created by a complete turn of the Himalayan vulture during thermal soaring (40 Hz tag attached with leg loop harness and equipped in France, see [53]) and (**b**) the ellipse mapped by a front-crawl double arm stroke of a human swimmer (40 Hz tag taped to the upper back aligned with the spine, equipped in Mallorca). Multiple examples of the two behaviours are shown with their centroids illustrated as vectors from the *m-sphere* origin. The *m-prints* of the vulture shift due to bank angle; where red and orange prints are performed with the opposing bank angle to blue and dark grey prints (clockwise and anti-clockwise turns). The dot product of the centroid vectors from that of the centroid of the NOP provides the average angle of bank in the roll axis adopted over a full turn. For the swimmer, a postural rotation in the double arm stroke creates the ellipse *m-print*, which moves about the NOP according to heading, hence the dot product of centroids in this case provide a heading (green prints have the opposite heading to orange prints)

Visualising TriMag data on the *m-sphere* for an extended period of time concatenates behaviours, producing a broad-brush picture of headings and postures, and potentially concealing valuable information on the sequence of rotations associated with a given behaviour. As with the calibration movements, a repetitive behaviour that involves stylised body-roll and/or body-pitch produces a specific trajectory of points on the *m-sphere*, here termed the ‘*m-print*’ (e.g. Fig. 8). The *m-print* can therefore be used to identify behaviour according to the conserved sequence of angular rotation, as well as to quantify consistency in behaviour as a measure of performance. Such behaviours can extend over time scales beyond those normally used in identification of behaviours via accelerometry (see examples below).

### Derivation of metrics from m-sphere plots

In addition, we propose a number of summary metrics that can be derived from isolated *m-prints*, which could be used as a basis for machine learning analogous to the summary metrics used to identify behaviour from acceleration data [40, 41]. We note that this is an introduction to potential TriMag derivatives, rather than an exhaustive list, as there is potential for more metrics to be derived, such as track tortuosity, which, while conventionally used for 2-dimensional movement, is likely to be useful in *m-sphere* representations.

#### Dot product metrics

As with the NOP, the position of the *m-print* may shift about the sphere according to the animal’s operational plane and general posture. It may therefore be appropriate to extract general postural information from the TriMag data (Williams et al., in preparation) to quantify variation between different cases of a single behaviour. This is derived from the centroid of the *m-print*, defined as the average location of its points of the sphere (Fig. 8), and equivalent to the mean or median values of its coordinates in each of the three axes. Taking a vector of this point from the origin of the sphere, the average posture can be calculated as the dot product (θ) between this vector and the centroid vector of the NOP (that which is equivalent to a level posture). Thus;

1 $$ A\cdot B=\left|\left| A\right|\right|\left|\left| B\right|\right| Cos\theta $$

Or

2 $$ \theta =\mathrm{acos}\left[\frac{\left( x\ast {x}_2+ y\ast {y}_2+ z\ast {z}_2\right)}{\sqrt{\left({x}^2+{y}^2+{z}^2\right)}\sqrt{\left({x_2}^2+{y_2}^2+{z_2}^2\right)}}\right] $$

where A and B are the vectors of the *m-print* and NOP centroids, respectively, and their corresponding coordinates are defined as (x, y, z) and (x_2_, y_2_, z_2_). The angle returned by this formula is a solid angle and is equal to the area of the segment of a unit sphere, centred at the angle’s vertex. This solid angle equals the area of a segment of a unit sphere in the same way that a planar angle equals the length of the arc of a circle [42].

In the case of the soaring vulture (Fig. 1), the TriMag data reveal the directional rotation of the bird where accelerometry data cannot. Defining a single turn by its *m-print*, the average body posture of the bird can be derived which, in this case, is its angle of bank (Fig. 8a). Bank angle modulates the lift that the bird experiences and therefore relates to soaring performance. Banked turns can also result in animals ‘pulling g’, whether in the air, water and on land. Such situations make TriMag data particularly relevant because acceleration data cannot be used to derive posture, when the combination of posture, speed and turn angle can represent interesting performance constraints [36]. The centroid approach can also be used to extract the average heading to examine directionality in behaviour (Fig. 8b).

#### Angular velocity metrics

We can calculate the angular velocity as a single value along the *m-print* that describes the trajectory of points in 3-dimensional space. The angular difference between successive data points is given as the dot product between the vectors from the origin of the *m-sphere* to each point (Eq. 2) with the angular velocity being this angle divided by time. Thus, for tags set to record at 40 Hz, as were most of those used in this study, an *m-sphere* angular velocity can be calculated up to every 25 milliseconds between successive data points.

**Fig. 9:**
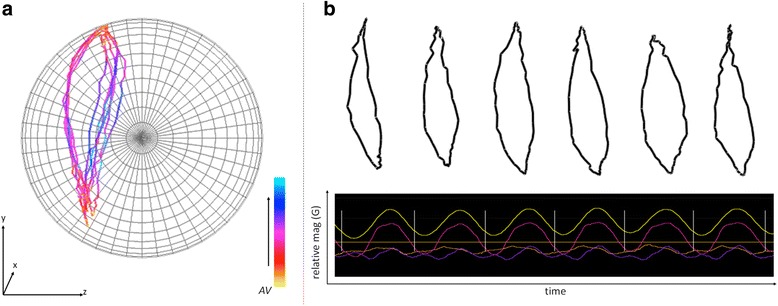
**a** TriMag data collected from a human front crawl swimmer in a sea-race (a back mounted tag attached with tape, sampling at 40 Hz). **b** The isolated *m-prints* for six double-arm strokes of the front crawl swim. The cases were separated using a Fast Fourier Transformation, FFT (*yellow waveform*) on the x-axis of the TriMag data (*pink waveform*). There is high consistency evident in the trajectory of points between the six cases, which for competitive swimmers is a measure of efficiency as they are not expected to show great variance across strokes although some of the observed differences may be due to the effects of wave chop

At once, calculation of the angular velocity highlights consistency between cases of a repetitive behaviour, especially considering the regularity of the actual form of the *m-print* combined with colour-defined levels of angular velocity (Fig. 9a). Using this approach, the turning behaviour of the vulture would be defined by a consistently low angular velocity over what would be a ring mapped on the sphere. The bio-logging-derived measure has value for multiple behavioural contexts. Examples include studies on navigation, where *m-print* metrics could highlight the circling movement sometimes seen in animals seeking to orientate themselves [43], or research into the acquisition of food or avoidance of predation where associated turns could be quantified [44]. Stemming from this and, given that there is a cost to turning that depends on the extent of the turn angle and the angular velocity [16, 45] we expect *m-print* angular velocity metrics to relate to movement energetics in the same way as have acceleration-derived metrics, such as VeDBA and ODBA [9, 46]. Importantly, *m-print* angular velocities will operate in 3-d magnetic space whereas studies conducted to date have only considered 2-d, horizontal, space [47].

#### Fast fourier transformation on metrics

**Fig. 10:**
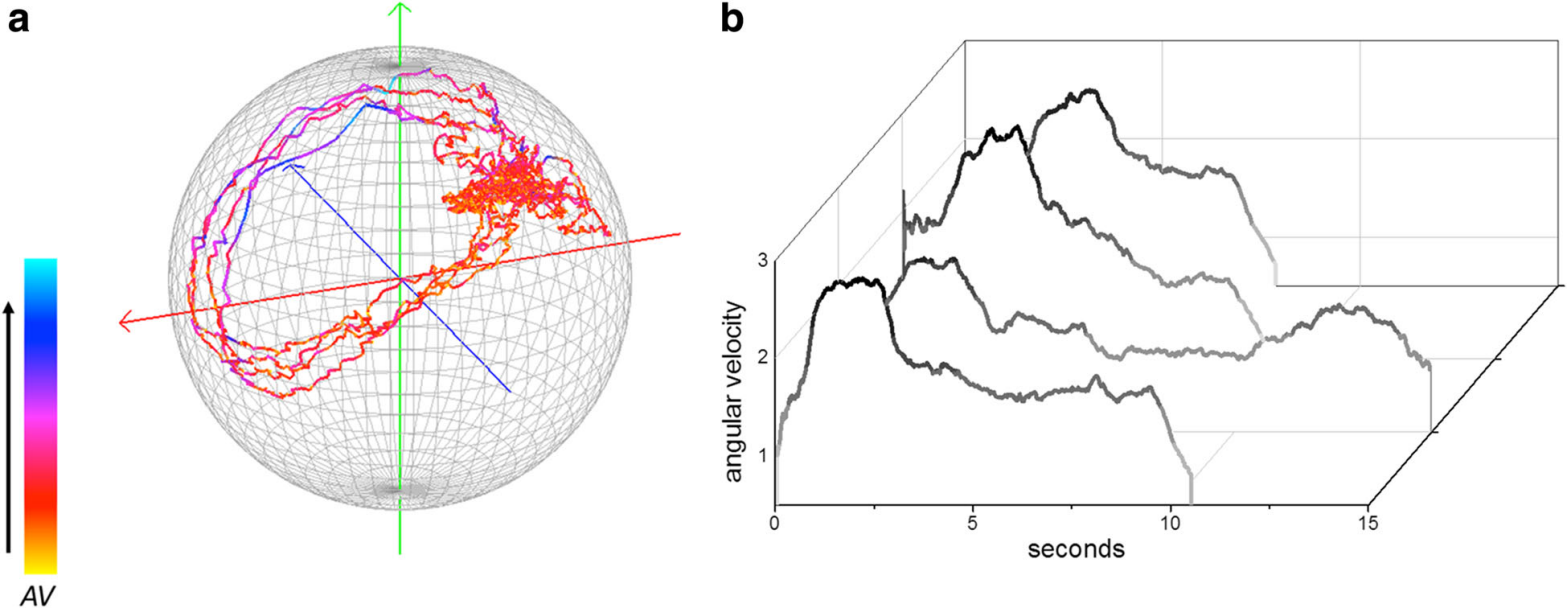
Four examples of the complete rotations seen in dynamic soaring of a wandering albatross (*Diomedea exulans*) (40 Hz tag attached to the back feathers with tape for a bird equipped on Marion Island), partitioned using FFT (isolated using in-house software Mk DD). **a** *M-sphere* with the four m-prints coloured by relative angular velocity (AV). **b** AV plotted by time for each of the four examples. Visualised in this way, the AV metric (relative to max. and min. values of data shown) can be used to quantify consistency in the trajectory of the behaviour and therefore performance

Isolating single *m-print* trajectories for a repetitive or periodic behaviour can be done manually, as was the case for the examples above, which is onerous, especially if the behaviour is short-lived. Alternatively, each case can be isolated using a Fast Fourier Transformation (FFT). The FFT has commonly been used to identify periodicity in behaviour and for example, applied to identify the frequencies of limb movement from acceleration data (e.g. in the Ethographer 1 software [48]) and periodic patterns in depth profiles such as those associated with diel vertical migration (e.g. [49]). Periodicity in behaviour measured with the TriMag should be readily identifiable with signal processing tools such as the FFT due to the low signal-to-noise ratio (Fig. 10b). This should allow periodic behaviours to be isolated, even where they do not result in change in either acceleration or environmental parameters (e.g. dive depth).

The FFT can be applied to isolate individual *m-print* trajectories automatically, using a zero-crossing approach to the inverse FFT to select complete oscillations i.e. complete cases for a repeated behaviour. Here, FFT is applied to extract the fundamental waveform and its low harmonics from the data recorded in a TriMag axis, before converting back to TriMag data using an inverse FFT. This removes any direct current components that create vertical offsets in the signal, forcing the waveform to oscillate about zero. Zero crossings (Fig. 10b, vertical lines) of the cyclical motion become easily defined and the *m-prints* can then be extracted as the data between crossings. We used this method to extract the *m-prints* associated with each stroke cycle performed by a human during a front-crawl sea swim (Fig. 9), as well as soaring cycles of a wandering albatross, *Diomedea exulans*, (Fig. 10) [50]. These examples encompass single behaviours (a stroke or a soaring cycle) that vary in duration.

Isolating prints with the FFT allows the consistency in angular velocity between complete cycles to be assessed. This is a key performance metric in sports. For instance, a high performing swimmer should show little variation around the maximal angular velocity through the stroke. Consistency in performance is also of interest in ecological contexts. For the albatross, this may provide insight into the development of dynamic soaring performance (*cf*. [51]), or the impact of changing aerial conditions on flight dynamics.

## Conclusion

The *m-sphere* is not a convenient artefact, but an inevitable consequence of plotting orthogonal magnetic field sensor data in tri-axial space. Similarly, *m-prints* on the *m-sphere* arise as a consequence of animal body rotations in any of the three space axes, and thus provide a picture of animal behaviour over time. Fundamental metrics derived from *m-prints,* such as individual *m-sphere* co-ordinates and angular velocity, reduce complex tri-axial data to two or one dimensions, facilitating interpretation. Given that magnetometers are sensitive to almost all rotational movement, TriMag information is particularly valuable for animals that operate in fluid media (air and water) where body pitch and roll angle frequently depart from the horizontal and co-vary with heading, providing ecological context to movement studies where interpretation of acceleration data can be limited.

Because magnetometers and accelerometers provide very different information regarding movement due to their different frames of reference, the future should see both sensors combined to create a powerful tool for accurately quantifying different aspects of movement. Acceleration-derived indices relating to the energetics of movement, such as VeDBA [52], could be enhanced by magnetometer equivalents (e.g. *m-sphere* angular velocity), relevant because there is an energetic cost to turning [47]. Enhancing the resolution of energetics together with behaviour in this way should strengthen our capacity to determine animal behaviours and their costs to a degree that was unthinkable just a few years ago.

## Additional file

Additional file 1: Comparison of tri-axial acceleration and tri-axial magnetometer data for detection of behaviours using template matching. (DOCX 20 kb)

## Abbreviations

FFT: Fast fourier transformation
G: Gauss
NOP: Normal operational plane
ODBA: Overall dynamic body acceleration
TriMag: Tri-axial magnetometry
VeDBA: Vectorial dynamic body acceleration
