# Supplementary material for: Identification of animal movement patterns using tri-axial magnetometry
Source: Mov Ecol. 2017 Mar 27;5:6. doi: 10.1186/s40462-017-0097-x (PMC5367006; doi:10.1186/s40462-017-0097-x)
Supplement: Additional file 1: — Comparison of tri-axial acceleration and tri-axial magnetometer data for detection of behaviours using template matching. (DOCX 20 kb) [file 40462_2017_97_MOESM1_ESM.docx]

**Additional file 1**

***Comparison of tri-axial acceleration and tri-axial magnetometer data for detection of behaviours using template matching.***

The three examples provided here are of behaviours with execution times between 2 and 20 s/cycle that are commonly performed by three species; a thermal-soaring vulture *Gyps himalayensis*, a washing penguin *Spheniscus magellanicus* and a swimming whale shark *Rhincodon typus*. We used the output of the TriMag sensor and acceleration sensors to template-match the axis that provided the clearest signature of each behaviours. For this, we used the time classifier software Framework4 (Walker *et al*. 2015) and tested the incidence of successful matches and the precision in classification of these behaviours.

The directional rotation associated with turning behaviour of the vulture in thermal soaring can be used to automatically classify behaviour by magnetometry data. Using a template of two full rotations in thermal soaring, classification by TriMag data (using the x-axis) had a much greater precision at 74% compared to that with acceleration where not a single template match was identified (using the same thresholds and scaling, Table) from a 78 minute file that was classified manually to contain 63 cases of the thermal soaring double rotation. A similar result was seen in the classification of swimming behaviour for the whale shark. Using a template of two body strokes in the z-axis, the proportion of positive classifications that were correct given manual classification was high for the magnetometer signal which, at 96%, correctly identified 417 of the 445 cases of the double stroke in 157 minutes of data recorded during diving (Table). The acceleration signal associated with the template only retrieved 1 match, which was correctly identified as the double body stroke.

Magnetometer and accelerometer data performed roughly equally well in the automatic classification of the alternate roll preening behaviour in the penguin. In a 808 minute data file, the behaviour of rolling to one side and then the other while washing with the flipper occurred 42 times according to manual classification. At a threshold of 70% correlation, classification by magnetometry classified 32 cases while the acceleration classified 18. However, precision was greater when the accelerometer data were used (86% precision compared to 50% precision, respectively).

| Species: behaviour template | Data length (min) | Template conditions | Total # cases | TriMag (TP/(TP+FP)) | TriAcc (TP/(TP+FP)) |
| --- | --- | --- | --- | --- | --- |
| Vulture: Soaring in thermals | 78 | 80% normalised correlation threshold  Scaling: 1/3, 1/2, 1/1, 2/1, 3/1  x-axes | 63 | 32/43 (74%) | 0/0 |
| Whale shark: Swimming | 157 | 80% normalised correlation threshold  Scaling: 1/2, 1/1, 2/1  z-axes | 445 | 417/435 (96%) | 1/1 |
| Penguin: Surface washing | 808 | 70% correlation threshold  Scaling: 1/1  z-axes | 42 | 32/64 (50%) | 18/21 (86%) |

Table 1: Classification of behaviours by template-matching using single axis outputs from magnetometer (TriMag) and accelerometer (TriAcc) sensors. Using the defined behavioural template for thermal soaring (vulture), swimming (whale shark) and surface washing (penguin), data files were manually classified to give the total number of cases for the behaviour. Directional rotation in thermal soaring and postural rotation in body strokes in swimming were defined by a single frequency sine wave template. Template-matching was therefore performed under normalised correlation with variable various scaling factors due to differences in behavioural duration. Preening by the penguin involved sine waves at two frequencies (one for the body roll and one for the flipper movements) and so a standard correlation template matching procedure was used. The number of positive matches differed between sensor types in the number of true positive (TP – cases correctly classified) and false positive (FP – cases incorrectly classified) results; summarised by their classification precision (given in %).

References:

Walker JS, Jones MW, Laramee RS, Holton MD, Shepard EL, Williams HJ, et al. Prying into the intimate secrets of animal lives; software beyond hardware for comprehensive annotation in “Daily Diary” tags. Mov Ecol. 2015;3(29).
